# Percutaneous Cholecystostomy Tube Leading to a “Floating” Gallbladder: A Case Report

**DOI:** 10.7759/cureus.5034

**Published:** 2019-06-29

**Authors:** Christopher Reilly, Saraswati Dayal, Chinwe Ogedegbe, Stephen Cohn, Javier Martin Perez

**Affiliations:** 1 Emergency Medicine / General Surgery, St. George's University School of Medicine, St. George, GRD; 2 Surgery, Hackensack University Medical Center, Hackensack, USA; 3 Emergency Medicine, Hackensack University Medical Center, Hackensack, USA; 4 Trauma, Hackensack University Medical Center, Hackensack, USA; 5 Surgery, Hackensack Meridian Health, Hackensack, USA

**Keywords:** gallbladder, ruptured, detached, floating, cholecystostomy, comorbid, elderly

## Abstract

In patients with significant comorbid conditions, acute cholecystitis is managed through surgical intervention or with cholecystostomy tube placement (CTP). The literature is not definitive in its recommendations for cholecystectomy versus cholecystostomy. This case report describes a presentation of acute calculous cholecystitis managed with CTP. Over a 10-week period, due to complications with the tube, the decision was made to perform a cholecystectomy. Upon open surgical exploration, an atraumatic, ruptured, and chronically inflamed gallbladder was found without attachment to the subhepatic plate and, in essence, free “floating” in the peritoneum. To our knowledge, this is the first-known documented case report in the English medical literature.

An elderly woman, with significant co-morbidities, following two months of antibiotic treatment for acute cholecystitis and subsequent percutaneous cholecystostomy tube placement and re-placements, underwent elective laparoscopic cholecystectomy, which was converted to open surgery. Upon exploration, a detached, “floating” gallbladder was found posterior to the transverse colon and removed after lysing extensive peritoneal adhesions. Subsequent to the cholecystectomy, the patient had uncomplicated recovery.

The literature does not present a clear consensus on CTP use vs early cholecystectomy in high-risk patients with acute cholecystitis. This management decision is based primarily on the surgeon’s clinical judgment and the use of evidence-based risk assessment indices. The "floating gallbladder" is a rare, benign complication that affirms the importance of extensively assessing the risks and benefits of CTP as compared to cholecystectomy in the elderly and/or comorbid patient.

## Introduction

Appropriate surgical intervention for cholecystitis is well-described in the literature, with an attendant low risk of complication. However, in complicated cholecystitis or in patients with an increased risk of comorbidity, the decision can be much more challenging. Percutaneous cholecystostomy tube placement (CTP) versus cholecystectomy in high-risk and/or elderly patients has been a topic of debate over the years in the surgical community, with some authors suggesting one as being superior to the other [1]. In reality, the decision is based primarily on the surgeon’s clinical judgment. Some literature suggests higher morbidity and mortality with CTP while others suggest that it is a useful permanent procedure in comorbid patients with acute cholecystitis [1-2]. To our knowledge, we describe the first case in the English medical literature of an episode of acute cholecystitis treated with CTP and, eventually, surgery, leading to the finding of an atraumatic, ruptured, and chronically inflamed gallbladder “floating” in the peritoneum. We believe this is a rare and interesting case due to the circumstances and findings.

## Case presentation

**Figure 1:**
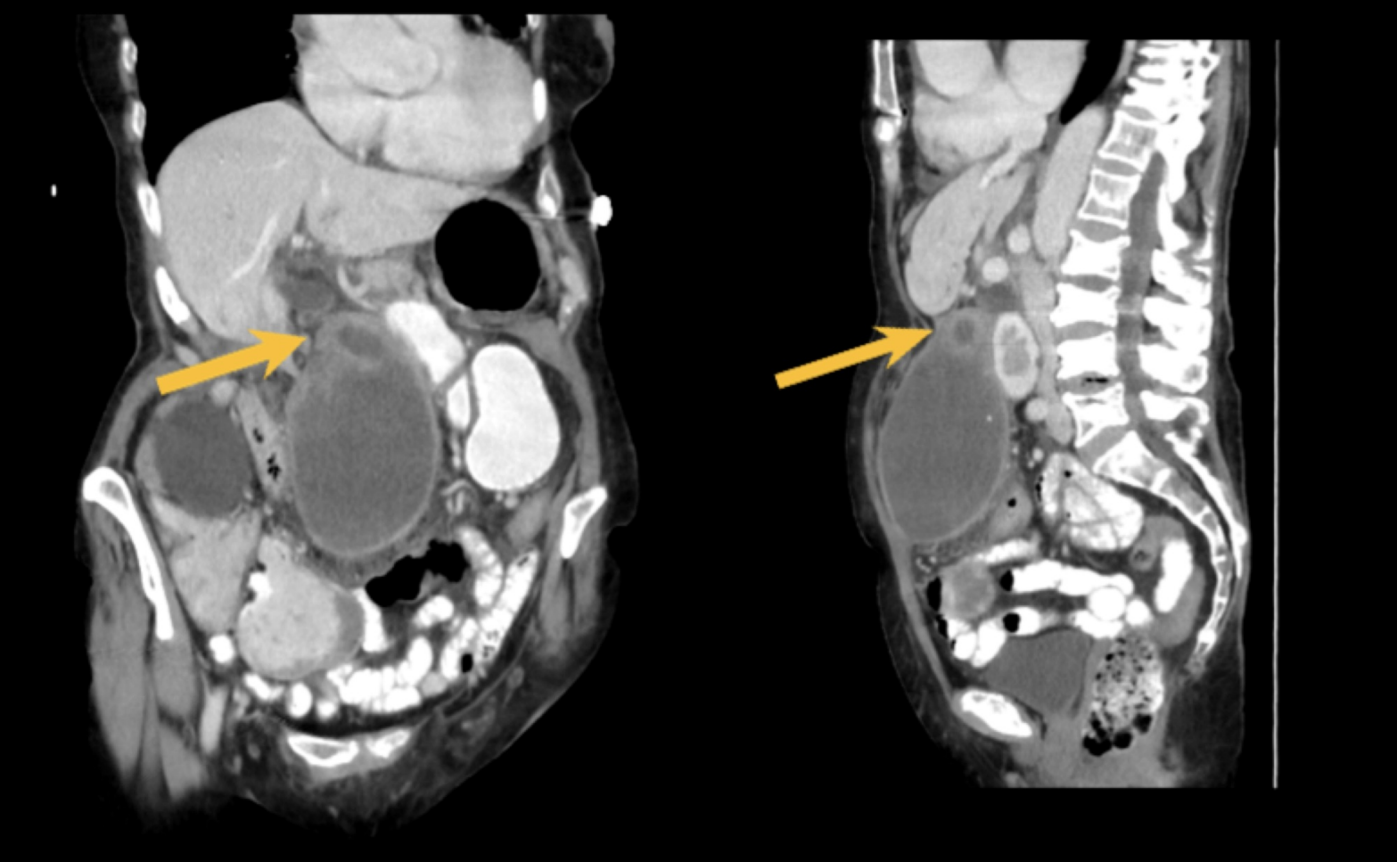
Left image: coronal section CT abdomen/pelvis with IV contrast, with the yellow arrow demonstrating a possible sight of gallbladder perforation/discontinuity; Right image: sagittal section of CT abdomen/pelvis with IV contrast, with the yellow arrow demonstrating possible perforation/discontinuity. CT = computed tomography; IV = intravenous

An 85-year-old Caucasian woman with no history of prior abdominal surgeries presented to the emergency department with three days of persistent cramping epigastric and periumbilical abdominal pain associated with constipation. Past medical history was significant for an L4 compression fracture, osteoporosis, hypertension, hypercholesterolemia, and asbestos pneumoconiosis, as well as a past surgical history of kyphoplasty and mastectomy. She had no known drug allergies and did not smoke, consume alcohol, or use illicit drugs. The patient’s medications include amlodipine, calcium and cholecalciferol, ondansetron, Percocet, Prolia, and simvastatin. On arrival, her vital signs were mildly abnormal, with a temperature of 99.9 F, blood pressure of 103/69 mmHg, heart rate of 114 beats per minute, respiratory rate of 18 breaths per minute, and oxygen saturation of 99% on room air. Initial evaluation in the emergency department included computed tomography (CT) imaging, which revealed gallbladder distention, wall thickening, and thin densities in the lumen, as seen in Figure 1.

This was diagnosed as acute cholecystitis and possible discontinuation of the gallbladder wall, suspicious for focal perforation. 

**Figure 2:**
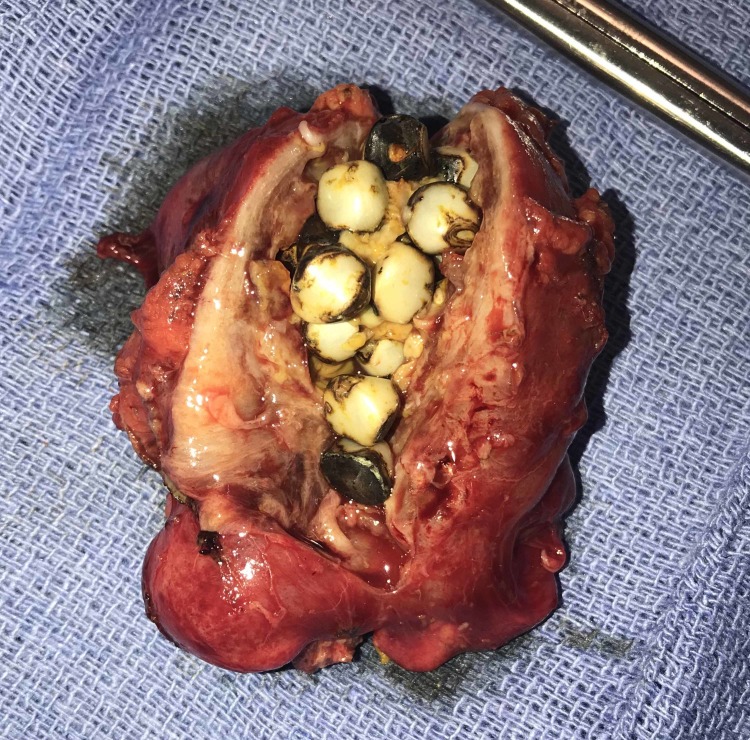
Gallstones ranging in size from 0.3 - 0.5 cm within the dissected gallbladder (post-cholecystectomy), which measured 5.5 cm in length and 3.5 cm in width.

Due to patient comorbidities, a decision was made to place a percutaneous cholecystostomy tube via interventional radiology (IR), as well as initiate antibiotic therapy. After this initial visit, the patient was discharged on antibiotics and about four weeks later, she was readmitted for an episode of Clostridium (C.) difficile colitis. At this time, she was also found to have her cholecystostomy tube dislodged and, subsequently, underwent replacement of the tube via IR. About four weeks later, following two additional tube dislodgements over an eight-day period, the decision was made to attempt laparoscopic removal of the patient’s gallbladder. In the operating room, when extensive intraperitoneal adhesions were encountered, the laparoscopic approach was converted to open cholecystectomy. During abdominal exploration, a firm mass was noted between the transverse colon and the left lobe of the liver. After extensive adhesiolysis, this mass was noted to have the appearance of a fibrotic and chronically inflamed gallbladder. There was no evidence of detachment prior to the surgery via imaging or physical exam (detachment, sepsis, etc.). This structure was removed from the peritoneal cavity and was opened to reveal 34 yellow/white/black gallstones, as seen in Figure 2.

The outer surface of the gallbladder was diffusely hemorrhagic, partially smooth and partially rough. The inner surface showed a yellow-tan irregular fibrotic surface with focal areas of hemorrhage and the thickness of the wall measured up to 1.7 cm. Pathology revealed areas of necrosis and infiltrates of pigment-laden and foamy macrophages suggestive of chronic inflammation, without any evidence of malignancy. The patient had an uneventful postoperative course and was discharged on postoperative day six.

## Discussion

This patient presented with multiple comorbidities, including her age, lung disease, and hypertension. Clinical judgment and risk analysis based on a Charlson Comorbidity Index score of 5 or a 21% 10-year survival rate (a score of 7 is a 0% 10-year survival rate) were used to assess this patient’s comorbidity status [3]. After weighing the score and clinical judgment, it was decided to perform a CTP rather than a laparoscopic abdominal surgery [3]. CTP has been known as an alternative treatment option that can be done safely at the bedside and without general anesthesia under ultrasound guidance via IR, as was done in this case [4]. Previous studies have shown the benefits of CTP in elderly and comorbid patients. According to one study that analyzed 278 consecutive patients who underwent CTP due to comorbidities, it was reported that there was a 5% 30-day mortality, and in 55% of the cases, CTP was the definitive treatment [1]. The literature also suggested that CTP under ultrasonographic guidance is a cost-effective, easy-to-perform, and reliable procedure with low complication and high success rates for critically ill patients [5]. According to another study, however, there is a high rate of recurrent biliary disease requiring intervention, suggesting routine interval cholecystectomy should be considered for the increased likelihood of biliary sepsis or other complications [6]. This suggests that CTP may not be a viable permanent solution in a high-risk category patient. This further suggests that these patients may eventually have an elective cholecystectomy performed to decrease the chance of further complication.

Recent literature documents that patients undergoing CTP were much more likely to have post-procedural infection (OR 2.25; 95% CI 2.07, 2.45), bleeding (OR 1.28; 95% CI 1.19, 1.37), and inpatient mortality (OR 9.27; 95% CI 7.95, 10.81), all with p-value <0.0001 (excluding shock and wound complications) [2]. On average, CTP patients’ length of stay (LOS) was 1.25 days longer after the procedure (95% CI 1.14, 1.37) [2]. Comparing post-procedural outcomes between CTP and cholecystectomy, it was found that elderly patients undergoing CTP had a higher rate of post-procedural morbidity and mortality [2]. CTP patients were more likely to have post-operative complications and were over nine times as likely to die during their course of hospitalization. This was attributed to cases of sepsis or tube dislodgement and hemorrhage [2]. This conflicts with many prior studies suggesting that CTP is safer for the elderly and/or comorbid over time. According to a Cochrane systematic review of randomized clinical trials, there was no significant difference seen in morbidity and mortality rates between the two interventions [7]. This was also seen in a retrospective single-institution study between drainage and surgical resection in high-risk patients regarding morbidity (17 vs. 9%, p = 0.67) and mortality (13 vs. 0%, p = 0.23), respectively [8]. It seems that the more recent data is more skewed towards surgical management [2], but given the lack of consensus among physicians and definitive evidence supporting certain criteria for selecting CTP over cholecystectomy in high-risk patients, this management decision is highly dependent on the individual physician’s clinical judgment.

In this case, the gallbladder neck was likely already fibrosed from extensive chronic inflammation of the cystic duct due to repeated bouts of cholecystitis. When examining the gallbladder, there were only the supersaturated stones present and no evidence of any bile; therefore, it is quite possible that the cystic duct was previously fibrosed. With inadequate drainage status post-CTP, the tube placement may have caused the gallbladder neck to partially separate from the subhepatic plate. The tube had become dislodged several times, inferring the possibility of an iatrogenic cause for the gallbladder detachment. This conservative decision to delay surgical intervention after multiple tube dislodgements was made based on the patient's multiple comorbidities (Charlson Comorbidity Scale) and no physical exam signs of gallbladder detachment or sepsis. There have been reports of CTP causing gallbladder wall necrosis following successful tube placement, which then progressed to sepsis and spontaneous rupture [9] but, on the contrary, this patient had no signs of sepsis, peritonitis, or spontaneous rupture. Another less likely reason for this abnormality could be that our patient may have had a congenital gallbladder anatomical variation. This possibility was very unlikely because when inspecting the gallbladder, there were no obvious anatomical or congenital anomalies present such as cystic duct aplasia, a variation in the location of the common bile duct, or gallbladder scleroatrophy [10]. At the time, there were no signs of gallbladder detachment and no imaging was done prior to surgical intervention, so it can only be surmised as to what caused this "floating" gallbladder presentation.

This presentation is important for surgeons and physicians to be aware of and consider when weighing the risks and benefits of performing CTP over cholecystectomy in high-risk patients. Typically, a ruptured gallbladder would present with positive peritoneal signs, sepsis, and/or even death, but on the contrary, this was a case where gallbladder detachment led to an asymptomatic presentation. This uncommon case demonstrates an interesting possibility that should be considered when evaluating cholecystitis in the highly comorbid/elderly patient. Following an extensive English medical literature search, we were unable to find a similar case.

## Conclusions

Currently, the literature does not present a clear consensus on CTP use vs early cholecystectomy in high-risk patients with acute cholecystitis. This management decision is based primarily on the surgeon’s clinical judgment. This clinical judgment is combined with other risk assessment tools, such as the Charlson Comorbidity Index, to come to a management decision. In our case of acute cholecystitis in the elderly and comorbid patient, it was decided to proceed with CTP. Over time and after multiple tube dislodgements, the decision was made to perform elective cholecystectomy. Upon surgical exploration, a chronically inflamed gallbladder was found without attachment to the sub-hepatic plate, "floating" in the peritoneum. Through this unique and first-known documented case presentation, we hope to increase awareness of the variety and complexity of complications associated with a long-term CTP as compared to early cholecystectomy.
